# Evaluation of Risk Factors for Vertebral Compression Fracture after Carbon-Ion Radiotherapy for Primary Spinal and Paraspinal Sarcoma

**DOI:** 10.1155/2017/9467402

**Published:** 2017-07-26

**Authors:** Yoshihiro Matsumoto, Makoto Shinoto, Makoto Endo, Nokitaka Setsu, Keiichiro Iida, Jun-Ichi Fukushi, Kenichi Kawaguchi, Seiji Okada, Hirofumi Bekki, Reiko Imai, Tadashi Kamada, Yoshiyuki Shioyama, Yasuharu Nakashima

**Affiliations:** ^1^Department of Orthopaedic Surgery, Graduate School of Medical Sciences, Kyushu University, 3-1-1 Maidashi, Higashi-ku, Fukuoka 812-8582, Japan; ^2^Ion Beam Therapy Center, SAGA HIMAT Foundation, Tosu, Japan; ^3^Research Center Hospital for Charged Particle Therapy, National Institute of Radiological Sciences, Chiba, Japan

## Abstract

**Background and Purpose:**

Carbon-ion radiotherapy (C-ion RT) was effective therapy for inoperable spinal and paraspinal sarcomas. However, a significant adverse event following radiotherapies is vertebral compression fractures (VCFs). In this study, we investigated the incidence of and risk factors for post-C-ion RT VCFs in patients with spinal or paraspinal sarcomas.

**Material and Methods:**

Thirty consecutive patients with spinal or paraspinal sarcomas treated with C-ion RT were retrospectively reviewed. Various clinical parameters and the Spinal Instability Neoplastic Score (SINS) were used to evaluate the risk factors for post-C-ion RT VCFs.

**Results:**

The overall incidence of VCFs was 23% (median time: 7 months). Patients with VCFs showed a markedly higher SINS score (median value, 9 points) than those without VCF (5 points). The area under the receiver operating characteristic curve for the SINS score was 0.88, and the optimum SINS cut-off score was 8 points. The cumulative incidence of VCFs at 1 year was 9% for patients with a SINS score under 8 points, versus 80% for those with a SINS score of 8 points or higher (*p* < 0.0001).

**Conclusions:**

In patients with a SINS score of 8 points or higher, referral to a spine surgeon for stabilization and multidisciplinary discussion is appropriate.

## 1. Introduction

Spinal and paraspinal sarcomas are rare tumors that are comprised 4% and 13% of bone and soft tissue sarcomas, respectively [1]. The optimal treatment of choice for spinal and paraspinal sarcomas is en bloc resection with wide margins [2]. However, en bloc resection of spinal and paraspinal sarcomas is very difficult or impractical because of the complexity of the surrounding anatomy [3, 4]. In cases where en bloc resection is not feasible, curettage or piecemeal excision may be applied, but both methods have always resulted in local recurrence.

Radiotherapy is an alternative option for patients with inoperable spinal or paraspinal sarcomas. Unfortunately, most sarcomas are radioresistant in nature, leading to a low probability of local control [5]. In recent decades, several new radiation modalities, such as carbon-ion radiotherapy (C-ion RT), have emerged. Compared to conventional photon radiotherapy, C-ion RT is considered to have greater relative biologic effectiveness (RBE) and better probability of achieving tumor control [6]. Consistent with this, several reports have proven that C-ion RT is a safe and effective modality for the management of high-grade sarcomas [7] and may be an effective alternative to surgery for inoperable spinal and paraspinal sarcomas [8].

In most cases of spinal and paraspinal sarcomas, the vertebral body and pedicle are destructed by the tumor mass. Furthermore, any radiotherapy modalities may cause soft mass shrinkage and bony necrosis of the vertebral body, leading to vertebral compression fractures (VCFs) and postradiation spinal deformities [9]. The risk of VCFs following radiotherapies, in particular stereotactic radiosurgery (SRS), has been reported in several studies. Rose et al. revealed that the rate of post-SRS VCFs was as high as 39% [10]. This high rate of VCFs should not be overlooked since patients who undergo radiotherapies are at risk of further surgical interventions to address treatment-induced VCFs. Thus, the potential risk factors for VCFs should be evaluated in every patient before administering radiotherapies. However, the incidence and risk factors for C-ion RT-induced VCFs have not been investigated thus far.

Recently, the Spine Oncology Study Group (SOSG) developed the Spinal Instability Neoplastic Score (SINS) to assess the degree of spinal stability caused by metastatic tumors [11]. The SINS has undergone extensive reliability testing, and excellent agreement was found among radiation oncologists, radiologists, and spinal surgeons for comparing spinal instability between stable and (impending) unstable cases [12]. However, this system has not been clinically validated in the field of primary spinal and paraspinal sarcomas. The objectives of this study were to ascertain the incidence of VCFs in patients with spinal or paraspinal sarcomas following C-ion RT and to determine the association between the SINS and other relevant clinical factors for predicting post–C-ion RT VCFs.

## 2. Materials and Methods

### 2.1. Patients

Thirty consecutive patients with spinal or paraspinal sarcomas who met all of the following eligibility criteria were retrospectively reviewed: histologically proven primary sarcoma; tumors judged to be medically inoperable by a multidisciplinary tumor board comprised of spine surgeons, radiation oncologists, neuroradiologists, pathologists, and oncologists; grossly measurable tumors <15 cm in greatest dimension; an Eastern Cooperative Oncology Group (ECOG) performance status from 0 to 2; no distant metastasis at initial referral for treatment; and no infection at the tumor site. Patients provided consent after being given the option to opt-out, and the study was approved by the institutional review board at the Kyushu University Hospital.

### 2.2. C-Ion RT

The C-ion RT technique was performed using approximately the same approach as reported previously [13, 14]. In brief, a set of 2 to 5 mm thick noncontrast-enhanced computed tomography (CT) images was obtained under respiratory gating for treatment planning purposes. The clinical target volume (CTV) usually included the potential area of tumor spread and was established as a 3–5 mm margin around the gross tumor. The planning target volume (PTV) added an additional 3–5 mm margin to the CTV, with modification to avoid severe toxicity, if the CTV was too close to critical organs, such as the spinal cord, the bowels, and the skin. Three-dimensional treatment planning for C-ion RT was carried out using the XiON software program (Elekta, Stockholm, Sweden; Mitsubishi Electric, Tokyo, Japan). The patients were positioned in customized cradles and immobilized with a low-temperature thermoplastic sheet. Dose was expressed as the relative biological effectiveness- (RBE-) weighted dose (Gy (RBE)), which was defined as the absorbed dose of carbon ions multiplied by the RBE [15]. C-ion RT was performed once daily, 4 days a week (Tuesday–Friday), for a total of 16 fixed fractions (fr.) over 4 weeks.

### 2.3. Radiographic and Clinical Assessment

After C-ion RT, patients were assessed clinically at 4–6 weeks posttreatment and then every 3 months thereafter. X-rays, MRI, CT, and PET-CT were used to evaluate radiological response. Local relapse (LR) was defined as progression based on tumor growth on imaging results and/or clinical symptoms such as worsening of pain or neurological symptoms. The exact pattern of LR, including within or adjacent to the irradiated field, was also evaluated. VCFs were defined as the development of de novo fractures or the progression of preexistent VCFs with relevant symptoms. To evaluate the risk factors for post-C-ion RT VCFs, each treated vertebral segment was scored according to the SINS criteria [11]. In particular, CT was used to categorize the type of bone lesion (i.e., lytic, sclerotic, or mixed). Spinal alignment was assessed based on X-ray, MRI, and CT imaging. To determine the vertebral component affected by the collapse and posterolateral tumor involvement, the T1-weighted MRI sequence was reviewed. A total score classified the patient as stable, potentially unstable, or unstable.

### 2.4. Statistical Analysis

Categorical variables were expressed as count and proportion, whereas continuous variables such as age and follow-up were expressed as mean ± standard deviation or median and range. The outcome variable of interest was the time to death, LR, or distant metastasis, and development of a de novo VCF or fracture progression. Overall survival (OS) and local control were estimated using the Kaplan-Meier method. The time to development of post-C-ion RT VCFs was calculated in months from the start date of C-ion RT or from the last follow-up imaging study if the patient was fracture-free. Death before fracture was considered a competing risk to fracture, and cumulative incidence rates of VCF were obtained using a competing risk model [11]. The log-rank test was used in univariate analysis to compare cumulative incidence rates of VCFs with a potential predictor of interest. A multivariate Cox proportional hazards regression model was applied to determine the joint effect of potential factors that were found significant on univariate analysis. Appropriate cut-off points for SINS scores and their ability to predict the occurrence of VCFs were obtained using receiver operating characteristic (ROC) curves corresponding to the point on the curve nearest the upper left corner of the ROC graph. Statistical significance was set at *p* < 0.05. JMP version 13 software was used for statistical analysis.

## 3. Results

### 3.1. Baseline Patient Demographic Characteristics

We retrospectively reviewed 30 patients with spinal (26 cases) or paraspinal (4 cases) sarcomas treated with C-ion RT. Patient characteristics are summarized in Table 1. The sample consisted of 18 men and 12 women, with a median age of 55.9 years (range, 20–82 years). Histological examination diagnosed the following types of spinal and paraspinal sarcomas: 8 cases of chordoma; 5 cases each of osteosarcoma, undifferentiated pleomorphic sarcoma, and malignant peripheral nerve sheath tumor; 2 cases each of chondrosarcoma and leiomyosarcoma; and 1 case each of myxofibrosarcoma, malignant solitary fibrous tumor, and malignant myoepithelioma. The ECOG performance status score was 0 or 1 in 27 patients and 2 in 3 patients. Tumor locations included the cervical vertebrae (*n* = 5), thoracic vertebrae (*n* = 7), lumbar vertebrae (*n* = 8), and sacral vertebrae (*n* = 10). Maximal lesion diameters ranged from 2.5 to 13 cm; the mean maximal diameter was 6.7 ± 2.8 cm. The maximal diameters of 19 cases were greater than 5 cm. The RT doses delivered were 57.6 Gy (RBE) (3.6 Gy (RBE)/fr.) in 1 patient, 64.0 Gy (RBE) (4.0 Gy (RBE)/fr.) in 3 patients, 67.2 Gy (RBE) (4.2 Gy (RBE)/fr.) in 8 patients, and 70.4 Gy (RBE) (4.4 Gy (RBE)/fr.) in 18 patients (mean dose: 68.5 Gy (RBE); median dose: 70.4 Gy (RBE)). The biological equivalent dose for photon therapy in 2 Gy fractions (EQD2) was calculated as fractionation varied using a linear quadratic formula with *α*/*β* of 10 Gy for tumor and *α*/*β* of 3 Gy for all organs at risk [16]. Various chemotherapy regimens were received by 16 patients before and/or after C-ion RT. The median follow-up was 20.5 months.

### 3.2. Overall Survival and Local Control

**Figure 1:**
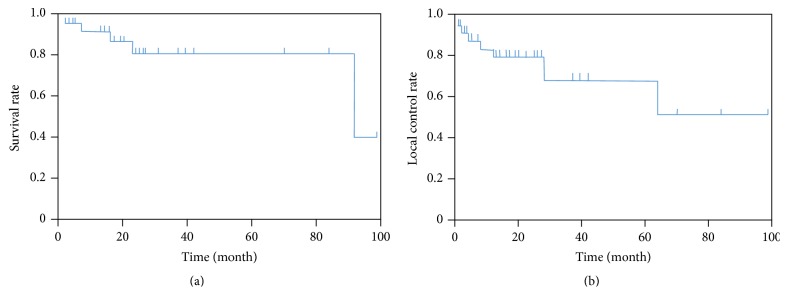
Kaplan-Meier survival curve for 30 cases with spinal or paraspinal sarcomas, showing overall survival (a) and local control (b) rates.

Five patients died over the entire follow-up period, all due to the initial sarcoma. The 2-year and 5-year overall survival (OS) rates were 81.8% and 81.8%, respectively (Figure 1(a)). Local relapse was observed in 7 patients (23%), with a median time to local relapse of 17 months (range, 3–69 months). The 2-year and 5-year local control rates were 80.1% and 70.8%, respectively (Figure 1(b)). Patterns of local relapse included progression within the irradiated field (2 of 7 [28.5%]) and progression at the margin of the irradiated field (5 of 7 [71%]). Management after local relapse consisted of decompression and stabilization surgery alone (2 of 7 [28.5%]), systemic chemotherapy alone (3 of 7 [43%]), or palliative care (2 of 7 [28.5%]). Distant metastases were observed in 9 patients (30%), most frequently in the lung (7 patients) and bone (4 patients). Overall, the 2-year and 5-year progression-free (PFS) survival rates were 65.9% and 33%, respectively.

### 3.3. Post-C-Ion RT VCFs

The overall incidence of VCFs was 23% (*n* = 7); 5 (71%) were de novo fractures and 2 (29%) were worsened preexisting fractures. No local relapse was observed before or at the time of VCFs. The mean and median times to fracture after C-ion RT were 8.4 months and 7 months, respectively (range, 3–19 months). The 6-month and 2-year cumulative incidence rates of VCF, defined as the proportions of patients with any VCFs, were 15% and 29%, respectively. All VCFs were observed inside the irradiated field during follow-up: 1, 3, and 3 involved the cervical, thoracic, and lumbar vertebrae, respectively. Of the 7 patients with VCFs, 2 (19%) were asymptomatic, while 5 (71%) experienced worsening pain after C-ion RT due to the VCFs. A dislocated VCF with associated neurological deficits was observed in 1 patient. Ultimately, 2 patients underwent salvage with instrumented spinal reconstructive surgery and 3 required medications for pain relief.

### 3.4. Evaluation of Risk Factors for Vertebral Compression Fractures

**Figure 2:**
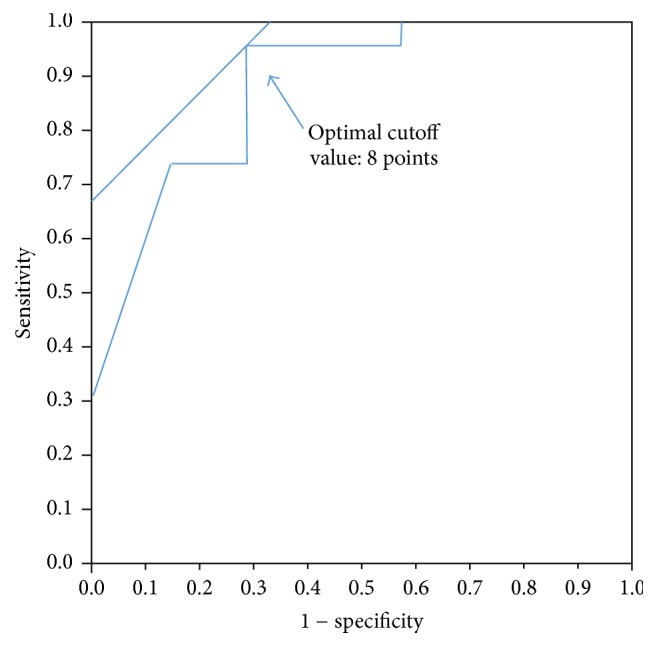
The optimum SINS cut-off score based on ROC curve analysis was 8 points.

**Figure 3:**
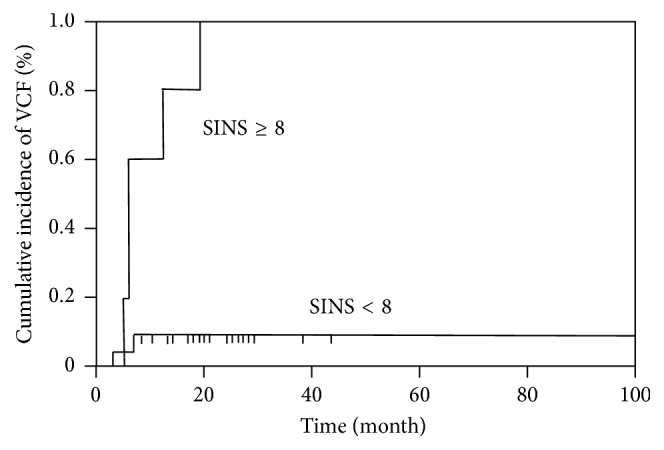
Cumulative incidence of vertebral compression fractures (VCFs) according to the SINS at 1 year.

A summary of the SINS criteria at baseline in patients with and without VCFs is shown in Table 2. Univariate analysis of each SINS component identified fracture location (junctional: *p* = 0.043), pain (mechanical: *p* = 0.043), alignment (not normal: *p* = 0.0006), and vertebral body collapse (positive: *p* = 0.0006) as predictors of VCFs after C-ion RT. However, none of these components reached statistical significance in multivariate analysis (data not shown). We then used the SINS to classify baseline patients as either stable (18 patients), potentially unstable (11 patients), or unstable (1 patient). Remarkably, only 1 patient classified as stable developed a VCF. Patients with a VCF had a markedly higher SINS score (median value, 9 points) than did patients without a VCF (median value, 5 points). The area under the ROC curve for the SINS score was 0.88, and the optimal SINS cut-off score was 8 points (Figure 2). Based on this cut-off value, the sensitivity and specificity for the prediction of VCFs were 96% and 71%, respectively. The 1-year cumulative incidence rates for any VCF were 9% for patients with SINS scores under 8 points versus 80% for patients with SINS scores of 8 points or higher (*p* < 0.0001) (Figure 3). Regarding additional factors, tumor size (>5 cm), total dose, chemotherapy, and patient age and sex were not significant predictive risk factors for post-C-ion RT VCFs in the univariate analysis (data not shown).

## 4. Case Presentation

**Figure 4:**
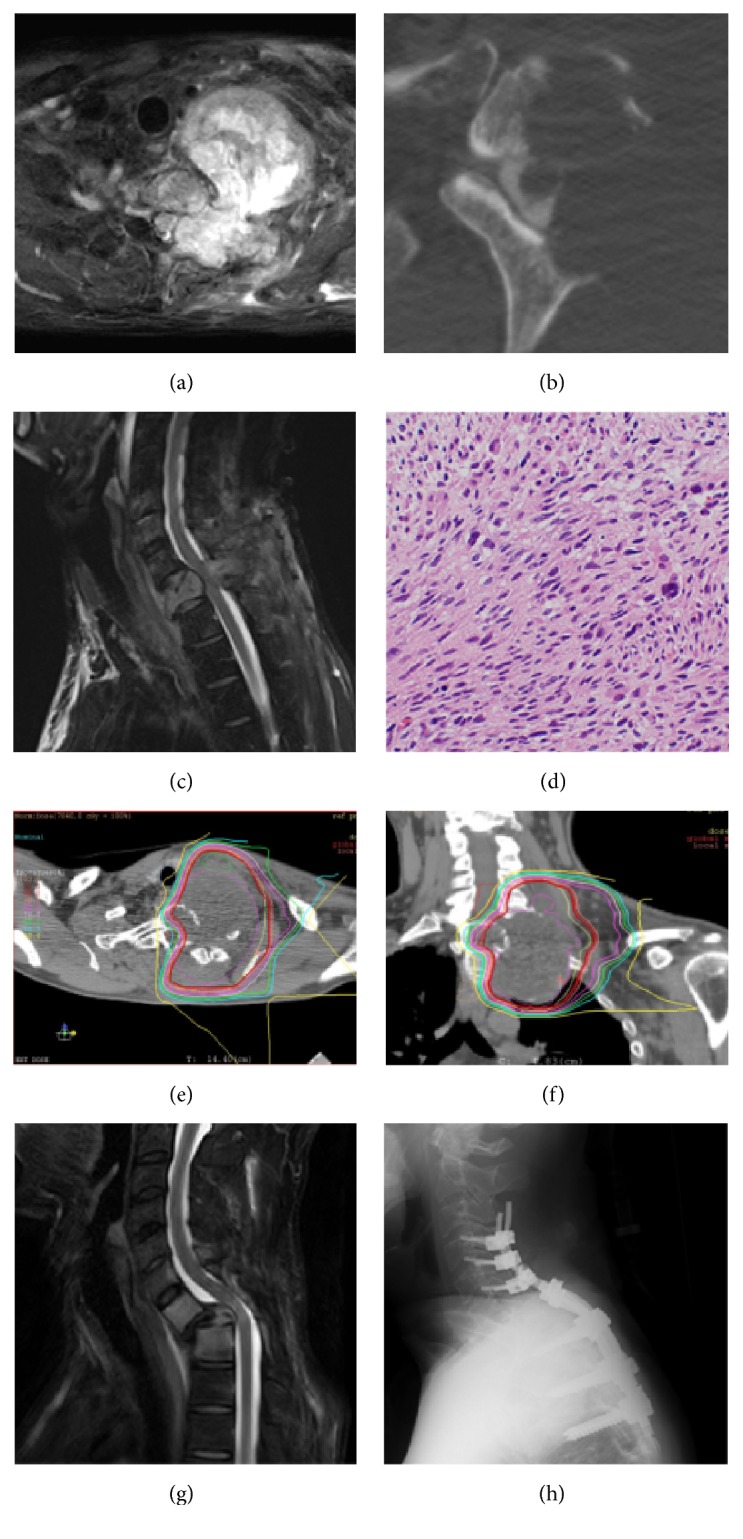
A 47-year-old man with a malignant peripheral nerve sheath tumor involving T1. (a) Axial T2-weighted MRI shows a huge, irregular, lobulated unilateral mass extending into the posterolateral component. (b) An axial myelo-CT scan shows the osteolytic mass. (c) Destruction of the vertebral body of T1 (20% collapse with kyphotic deformity) is also seen on sagittal T2-weighted MRI. (d) Histological examination shows the proliferation of oval to spindle cells arranged in a fascicular pattern with nuclear atypia and pleomorphism, confirming the diagnosis of malignant peripheral nerve sheath tumor (H&E, original magnification ×200). ((e) and (f)) Axial (e) and coronal CT (f) slices showing the dose distribution indicate the highly conformal nature of the C-ion RT. (g) Sagittal T2-weighted MRI at 5 months after C-ion RT (70.4 Gy (RBE)) demonstrates progression of the vertebral collapse and dislocation at the T1/2 level. (h) The patient underwent a posterior spinal fusion with instrumentation.

A 47-year-old man suffered from neck and left shoulder pain. Axial T2-weighted MRI showed a huge, irregular, lobulated unilateral mass extending into the posterolateral component (Figure 4(a)). An axial myelo-CT scan showed an osteolytic mass (Figure 4(b)). Destruction of the T1 vertebral body (20% collapse with kyphotic deformity) was seen on sagittal T2-weighted MRI (Figure 4(c)). The SINS score in this case was 13 points, and the patient was classified as unstable. Histological examination showed the proliferation of oval to spindle cells arranged in a fascicular pattern with nuclear atypia and pleomorphism, confirming the diagnosis of malignant peripheral nerve sheath tumor (Figure 4(d)). C-ion RT was performed with 70.4 Gy (RBE) in 16 fractions. The CTV was established as a 3 mm margin around the gross tumor volume. The T1 vertebral body with tumor invasion was also included in the CTV. The PTV added an additional 3 mm margin for possible positioning errors, respecting anatomic boundaries such as the spinal cord, the esophagus, the trachea, and the skin. Regarding the T1 vertebral body dose-volume parameters, *D*_max_ (the maximal absolute dose), *D*_90_, and *D*_50_ (the absolute doses covering 90% and 50%, resp.), and *V*_50_ and *V*_80_ (the relative volumes receiving more than 50% and 80% of the total dose, resp.) were 70.6 Gy (RBE), 33.9 Gy (RBE), 67.0 Gy (RBE), 89%, and 67%, respectively (Figures 4(e) and 4(f)). Five months after C-ion RT, the patient felt weakness in the lower legs bilaterally, and follow-up sagittal T2-weighted MRI demonstrated progression of vertebral collapse and dislocation at T1/2 (Figure 4(g)). The patient underwent a partial laminectomy and posterior spinal fusion with instrumentation (Figure 4(h)) and was subsequently treated by chemotherapy. However, multiple lung and bone metastases developed and the patient died 17 months after C-ion RT.

## 5. Discussion

The development of VCFs following radiotherapy has only recently become a major concern as a significant, common adverse event [17]. Osteoradionecrosis is the proposed pathogenesis, with the hypothesis that both vertebral bone and tumor tissue are replaced with necrotic fibrous tissue after radiation, resulting in collapse of the vertebral body. Although the risk of VCF is about 5% after conventional photon radiotherapy, the crude risk of SRS-induced VCFs has been shown to range from 11% to 39% [18, 19]. Since C-ion RT has greater RBE than SRS, the risk of post-C-ion RT VCFs might be higher than that of VCFs associated with SRS. However, the incidence of and risk factors for post-C-ion RT VCFs have not been described, and as such this has been an urgent clinical problem.

The incidence of postradiation VCFs may depend on preexisting spinal instability. Recently, the SOSG developed the SINS, a reliable scoring system to detect spinal instability [11]. The clinical validation of the SINS has been limited to selected case series [19, 20]. For example, Sahgal et al. investigated the risk factors for VCFs using the six SINS criteria. Preexisting VCFs, bone lesion type (lytic tumor), and malalignment were found to be significant factors, indicating the utility of the SINS in predicting the postradiotherapy risk of VCFs [18].

In this study, we report the first clinical observation of an association between the SINS criteria and post-C-ion RT VCFs in patients with spinal or paraspinal sarcomas. Univariate analysis suggested that fracture location (junctional), pain (mechanical), alignment (not normal), and vertebral body collapse (positive) were risk factors for post-C-ion RT VCFs. These findings are essentially consistent with those of other studies. Importantly, none of the SINS components individually were predictive in multivariate analysis. However, a larger sample size is required before solid conclusions can be drawn with respect to the utility of any single SINS component in predicting the risk of post-C-ion RT VCFs.

In contrast, we did find that the overall SINS score was useful for predicting the risk of post-C-ion RT VCFs. The 1-year cumulative incidence rate of any VCF was 80% for patients with a SINS score of 8 or higher. Such a significant incidence of post-C-ion RT VCFs in high-risk patients raises the question of possible interventions to prevent VCFs. Thus, it is strongly recommended that after C-ion RT, all patients with a SINS score of 8 or higher consult with a spine surgeon for stabilization and multidisciplinary discussion, as in the case of metastatic spinal tumors [21].

Although this study demonstrated a clinical association between the SINS and post-C-ion RT VCFs, there are several study limitations. First, scoring of the pain component may not have been completely reliable because of the retrospective nature of the study. The SINS defines mechanical pain as movement-evoked pain that is relieved with recumbency [21]. Over- or underestimation of pain may have occurred, which would have influenced the overall SINS scores. Second, the SINS was not externally validated in the original study, even though excellent inter- and intraobserver reliability were demonstrated [11]. To overcome these shortcomings, we are planning a prospective study to further validate the SINS in this field.

In conclusion, use of the SINS in clinical settings may significantly improve decision-making processes with regard to predicting post-C-ion RT VCFs. Patients with a SINS score of 8 points or higher should ideally be referred to a spine surgeon for stabilization as well as multidisciplinary discussion. If the score is low (0–7 points) and the patient shows no symptoms, prompt surgery may be avoided and conservative treatment is a reasonable option.

## Figures and Tables

**Table 1:** Patient characteristics.

Variable	Number of cases
Total patients	30
Sex	
M	18
F	12
Level	
Cervical	5
Thoracic	7
Lumbar	8
Sacral	10
Histology	
Chordoma	8
Osteosarcoma	5
UPS^*∗*^	5
MPNST^*∗∗*^	5
Chondrosarcoma	2
Leiomyosarcoma	2
Others	3
Tumor size	
<5 cm	
≤5 cm	
Irradiation dose	
<70.4 Gy (RBE)	18
70.4 Gy (RBE)	12
Chemotherapy	
Yes	16
No	14

^*∗∗*^MPNST: malignant peripheral nerve sheath tumor; ^*∗*^UPS: undifferentiated pleomorphic sarcoma.

**Table 2:** Baseline SINS classification according to VCF status.

SINS component	VCF (*n* = 7)	No VCF (*n* = 23)
Location		
Junctional	3	4
Mobile spine	3	5
Semi-rigid	1	3
Rigid	0	11
Pain		
mechanical	5	2
occasional and nonmechanical	2	13
pain free	0	8
Bone lesion		
lytic	5	19
mixed	2	4
blastic	0	0
Radiographic spinal alignment		
subluxation or translation	0	0
kyphosis or scoliosis	3	0
normal	4	23
Vertebral body collapse		
≥50%	0	0
<50%	2	1
no collapse but >50% involved	4	14
none of the above	1	8
Posterolateral involvement of spinal element		
bilateral	0	2
unilateral	7	15
none of the above	0	6
SINS classification		
unstable (score of 13–16)	1	0
potentially unstable (7–12)	5	6
stable (0–6)	1	17
